# High-Fidelity Modelling Methodology of Light-Limited Photosynthetic Production in Microalgae

**DOI:** 10.1371/journal.pone.0152387

**Published:** 2016-04-07

**Authors:** Andrea Bernardi, Andreas Nikolaou, Andrea Meneghesso, Tomas Morosinotto, Benoît Chachuat, Fabrizio Bezzo

**Affiliations:** 1 CAPE-Lab (Computer-Aided Process Engineering Laboratory) and PAR-Lab (Padova Algae Research Laboratory), Department of Industrial Engineering, University of Padova, Padova, Italy; 2 Centre for Process Systems Engineering, Department of Chemical Engineering, Imperial College London, London, United Kingdom; 3 PAR-Lab (Padova Algae Research Laboratory), Department of Biology, University of Padova, Padova, Italy; University of Nottingham, UNITED KINGDOM

## Abstract

Reliable quantitative description of light-limited growth in microalgae is key to improving the design and operation of industrial production systems. This article shows how the capability to predict photosynthetic processes can benefit from a synergy between mathematical modelling and lab-scale experiments using systematic design of experiment techniques. A model of chlorophyll fluorescence developed by the authors [Nikolaou et al., J Biotechnol 194:91–99, 2015] is used as starting point, whereby the representation of non-photochemical-quenching (NPQ) process is refined for biological consistency. This model spans multiple time scales ranging from milliseconds to hours, thus calling for a combination of various experimental techniques in order to arrive at a sufficiently rich data set and determine statistically meaningful estimates for the model parameters. The methodology is demonstrated for the microalga *Nannochloropsis gaditana* by combining pulse amplitude modulation (PAM) fluorescence, photosynthesis rate and antenna size measurements. The results show that the calibrated model is capable of accurate quantitative predictions under a wide range of transient light conditions. Moreover, this work provides an experimental validation of the link between fluorescence and photosynthesis-irradiance (PI) curves which had been theoricized.

## 1 Introduction

Microalgae are considered one of the most promising feedstocks for medium-term biofuel production [1], and have been the subject of extensive research in recent years. Despite this recognised potential, one of the main issues to address is bridging the gap between maximal theoretical biomass productivity (or even lab-scale realised productivity) and the actual biomass productivity in large scale production systems. To meet this objective, models capable of reliable and quantitative prediction of all key phenomena affecting microalgae growth, particularly light utilization, can be a great help to improve our understanding as well as optimise process design and operation [2, 3].

Quite a large number of modelling approaches have been proposed over the years, and it is beyond the scope of this article to provide an exhaustive review of the literature. The focus here is on the so called state models, based on the concept of photosynthetic unit (PSU), which have proved especially useful to characterise photosynthetic operation in practice. Kok [4] first published a state model describing the damage of the photosynthetic apparatus under high light conditions and the subsequent repair processes occurring in dark conditions. Later, Eilers and Peeters [5] proposed a state model that predicts steady-state photosynthetic productivity, also considering the inhibitory effect of excess light. Wu and Merchuck [6] extended Eilers and Peeters’ model by introducing a maintenance term representing the loss of biomass due to respiration when the culture is kept in the dark. The model by Han et al. [7] is equivalent to the model by Eilers and Peeters, but it refines the biological interpretation of the model parameters. In the work by Rubio et al. [8] the effect of photoacclimation is also considered, that is the ability of microalgae to adjust their pigment content and composition under varying light and nutrient conditions. Photoacclimation was first represented as a steady state process, then the model was later extended to represent photoacclimation dynamics [9]. Likewise, Nikolaou et al. [10] have recently proposed a model building on both Han and Droop models to describe the dynamics of photoproduction, photoinhibition and photoacclimation. Multiphysics models that integrate these biological state models within computational fluid dynamics simulation in order to represent the effect of light attenuation and mass-transfer limitation, have also started to emerge [11, 12]. Dynamic models for long-term algae bioprocess simulation in both laboratory scale system [13] and industrial-scale system [14], which incorporate more operating factors (light attenuation, nutrient concentration, temperature, etc.), can also be found in the literature.

Recently, Nikolaou et al. [15] have proposed a semi-mechanistic model capable of quantitative prediction of the flux of fluorescence by taking into account three distinct processes acting on different time scales: photoproduction encompassing all the processes that are responsible for the capture and utilization of photons; photoinhibition, the observed loss of photosynthetic productivity as a result of excess or prolonged exposure to light that is associated with the damage of functional components of the photosynthetic apparatus; and photoregulation, also referred to as non-photochemical-quenching (NPQ), encompassing all the regulatory mechanisms that dissipate excess excitation energy as heat. It was established through this work that the dynamics of chlorophyll-a fluorescence can help provide good predictions of the photosynthetic response under variable light conditions, thus allowing for the mathematical modelling of key photosynthetic mechanisms. However, it has recently been found that this fluorescence model may not describe photoregulation adequately over long-term experiments, thus advocating for a more detailed and biologically consistent representation.

The development of reliable mathematical models presents many challenges, particularly when multiple phenomena spanning several orders of magnitude both in time and space scales are to be accounted for, as is the case in [15]. This results in a number of identifiability (and therefore experimental) issues [16]. For the purpose of design, control and optimisation nonetheless, model identification is key to attributing a biological meaning to the parameters as well as to enabling accurate predictions under conditions that differ from those used during the identification experiments (extrapolation) [17].

Experiments are often designed and planned based on the available biological insight about the system, but as the complexity of phenomena and their correlation (as typically occurs in multiscale systems) increases, an experimenter’s expertise alone may no longer be sufficient to identify a model in a statistically meaningful way. Accordingly, more informative experimental protocols are required, as determined for instance with an optimal design of experiments approach [18]. In particular, model-based design of experiments (MBDoE) provides a systematic approach to determining an experimental protocol that would maximise the level of information available for identifying that model [18]. This approach has been successful in numerous applications, including biological systems [19, 20].

The core of this contribution is to demonstrate how our understanding and prediction capability of photosynthetic process may benefit from a synergy between modelling and experiments using MBDoE. As a case study we consider the model by [15] as a starting point. Experimental evidence [21] suggests that photoregulation mechanisms involve at least two interdependent processes, which calls for a more complex NPQ representation in this model. At the same time, a more complex NPQ representation augments the number of unknown parameters, a condition that we will show can be compensated by a wider array of experimental measurements combined with MBDoE. Eventually, we will be able to propose a biologically consistent description of the NPQ mechanism leading to a significantly improved prediction capability of photosynthetic production under a great variety of light conditions. Finally, we will be able to verify the link between fluorescence and photosynthesis-irradiance (PI) curves experimentally, a link that was theoricized in [22].

The remainder of the article is organised as follows. Section 2 presents the experimental methods and protocols used to develop and identify the proposed model. The dynamic model of chlorophyll fluorescence is presented and discussed in Section 3.1, with a particular focus on the description of NPQ activation/relaxation. This new model calls for a tailored calibration procedure in order to distinguish between the dynamics of NPQ and photoinhibition, whose time-scales are overlapping. The model’s identifiability is analysed in Section 3.2. Section 4 applies this methodology to accurately calibrate and validate the model for the microalga *N. gaditana*. A first calibration of the model parameters is conducted in Section 4.1, followed by a sensitivity analysis and model-based design of experiment in Section 4.2 and 4.3, respectively. The calibration results are discussed in Section 4.4, and the model is validated against multiple additional experiments in Section 4.5. Finally, Section 5 concludes the paper. Symbols and acronyms used in this work are listed and defined in Table 1.

**Table 1:** Symbols and acronyms. meaning.

Symbol	Meaning and units
*α_F_*	activity level of fast energy dependent quenching [-]
*α_S_*	activity level of slow energy dependent quenching [-]
*α_SS_*	reference function for energy dependent quenching activity [-]
*A*	fraction of photosynthetic units in open state
*B*	fraction of photosynthetic units in closed state
*C*	fraction of photosynthetic units in inhibited state
*η_D_*	rate of basal termal decay relative to the rate of fluorescence [-]
*η_I_*	rate of inhibition related quenching relative to the rate of fluorescence [-]
*η_P_*	rate of photoproduction relative to the rate of fluorescence [-]
*η_q_E*	rate of energy dependent quenching relative to the rate of fluorescence [-]
${\bar{\eta }}_{\text{qE}}^{C}$	maximum rate of interaction energy dependent quenching relative to the rate of fluorescence [-]
${\bar{\eta }}_{\text{qE}}^{F}$	maximum rate of fast energy dependent quenching relative to the rate of fluorescence [-]
${\bar{\eta }}_{\text{qE}}^{S}$	maximum rate of slow energy dependent quenching relative to the rate of fluorescence [-]
*F*′	maximum rate of slow energy dependent quenching relative to the rate of fluorescence [-]
*F_0_*	dark-adapted minimal fluorescence flux
$F_{0}^{\prime }$	light-adapted minimum fluorescence flux
*F_m_*	dark-adapted maximal fluorescence flux
$F_{m}^{\prime }$	light-adapted maximum fluorescence flux
$\Phi_{f}^{A}$	fluorescence quantum yield of a reaction centre in state A [-]
$\Phi_{\text{P}}^{A}$	quantum yield of photosynthesis of an open reaction centre of the photosystem II [$\mu {\text{mol}}_{{\text{e}}^{-}}\;\mu {\text{E}}^{-1}$]
$\Phi_{f}^{B}$	fluorescence quantum yield of a reaction centre in state B [-]
$\Phi_{f}^{C}$	fluorescence quantum yield of a reaction centre in state C [-]
$\Phi_{f}^{C}$	fluorescence quantum yield of a reaction centre in state C [-]
Φ*_f_*	quantum yield of fluorescence [−]
Φ*_P_*	photosynthesis quantum yield [$\mu {\text{mol}}_{{\text{O}}_{2}}\;\mu {\text{E}}^{-1}$]
Φ_PS2_	realised quantum yield of photosynthesis [$\mu {\text{mol}}_{{\text{e}}^{-}}\;\mu {\text{E}}^{-1}$]
*I*_qE_	irradiance level at which half of the maximal qE activity is realised [*μ*Em^−2^s^−1^]
*k_d_*	damage rate constant [-]
*k_r_*	repair rate constant [s^−1^]
*N*	chlorophyll specific number of photosynthetic units [$\mu {\text{mol}}_{O_{2}}\;{\text{g}}_{\text{chl}}^{-1}$]
*n*	Hill parameter related to the shape of sigmoid function describing NPQ activity [-]
*ν*	number of electrons per molecule of dissociated H_2_O
*P*	photosynthesis rate [$\mu {\text{mol}}_{O_{2}}\;{\text{g}}_{\text{chl}}^{-1}\;s^{-1}$]
qNPQ	NPQ index [-]
*S_F_*	scaling factor for fluorescence model (proportional to the chlorophyll content)
*σ*	total cross section [$m^{2}{\text{g}}_{\text{chl}}^{-1}$]
*σ*_PS2_	effective cross section of photosystem II [m^2^ *μ*E^−1^]
*τ*	turn over rate [s]
*ξ_F_*	time constant of the fast NPQ activation/relaxation mechanism [s^−1^]
*ξ_S_*	time constant of the slow NPQ activation/relaxation mechanism [s^−1^]
Acronym	Meaning
ASII	functional antenna size
DCMU	3-(3,4-dichlorophenyl)-1,1-dimethylurea
MBDoE	model-based design of experiments
NPQ	non-photochemical-quenching
PAM	pulse amplitude modulation
PI	photosynthesis-irradiance
PSII	photosystems II
PSU	photosynthetic unit
RCII	reaction centre of photosystem II

## 2 Material and Methods

### 2.1 Strain Cultivation

Microalgae *Nannochloropsis gaditana* (CCAP, strain 849/5) were grown in a sterile, filtered F/2 medium, using sea salts (32 gL^−1^) from Sigma, 40 mMTris HCl, pH 8 and Sigma Guillard’s (F/2) marine water enrichment solution. Growth experiments were performed in the multi-cultivator MC 1000-OD system (Photon Systems Instruments, Czech Republic) at a temperature of 21°C and a light intensity of 100 *μ*Em^−2^s^−1^ provided continuously by an array of white LEDs. The suspension culture was constantly mixed and aerated by bubbling air. Pre-cultures were grown at 100 *μ*Em^−2^s^−1^ in glass bottles of 0.25 L under a continuous airflow, enriched with 5% CO_2_. At the exponential phase, the pre-culture was centrifuged and re-suspended in fresh medium to reach a final concentration of 9 · 10^6^cellmL^−1^, before introduction in the multi-cultivator.

Three types of measurements are used for model validation and calibration purposes later on, which are described in the following subsections.

### 2.2 PAM fluorometry

When a sample containing microalgae is exposed to light, a fraction of the incoming photons is absorbed by pigment molecules, another fraction is scattered out and the rest passes through. The absorbed photons have three possible fates: they can either be captured by the reaction centre of photosystem II (RCII) to drive photosynthesis (photoproduction), or dissipated as heat (photoregulation), or re-emitted as fluorescence [23]. Thus, much information about the photosynthetic processes can be inferred by measuring the fluorescence fluxes under specific lighting protocols that preferentially activate or inactivate the photoproduction and photoregulation mechanisms.

Today’s state-of-the-art equipment, such as pulse amplitude modulation (PAM) fluorometers, can implement complex light protocols and record fluorescence fluxes both fast and accurately. PAM fluorometry excites microalgae photosynthetic apparatus by using three distinct light sources: a weak modulated light to measure the fluorescence flux; an actinic light to drive photosynthesis by exciting the photosynthetic apparatus and activating photoregulation; and a light pulse of high intensity to saturate the photosystems II (PSII).

Depending on the light conditions, the following fluorescence fluxes can be recorded by a PAM fluorometer [24]:

- *Dark-adapted, minimum fluorescence flux*, *F*_0_: The measuring light is applied to a sample that has been kept in the dark a sufficient length of time to completely oxidize the RCII. In the dark adapted sample the active RCII are thus ready to accept incoming photons (open state) and the NPQ processes are inactive.
- *Dark-adapted, maximum fluorescence flux*, *F*_m_: The measuring light is applied, after a short and intense light pulse, to a dark adapted sample. The PSII are completely reduced after the intense light pulse, so the active RCII are all occupied and cannot accept further photons (closed state), while the NPQ processes remain inactive.
- *Light-adapted, minimum fluorescence flux*, $F_{0}^{\prime }$: The measuring light is applied to a sample that has been exposed to a constant actinic light, after switching-off of the actinic light. The PSII are completely oxidized and the NPQ processes are active due to the application of an actinic light.
- *Light-adapted, maximum fluorescence flux*, $F_{m}^{\prime }$: The measuring light is applied, after a short and intense light pulse, to a sample that has been exposed to a constant actinic light. The PSII are completely reduced and the NPQ processes are active.
- *Light-adapted, steady-state fluorescence flux*, *F*′: The measuring light is applied to a sample that has been exposed to a constant actinic light. The PSII are only partially reduced, meaning that the active RCII are in a mix of open and closed states and the NPQ processes are active.

All of the above-mentioned fluorescence fluxes also depend on the presence of damaged (inactive) RCII that result from the evolution of photoinhibition when the incoming photons flux exceeds the capacity of the PSII.

In this work, all the fluorescence measurements are performed using a Dual PAM (Walz, Germany), after a dark adaptation period of 20 min. For model calibration purposes three fluorescence experiments are performed. The first experiment (Exp1) considers a light protocol with increasing actinic light intensities applied in steps of 60 s. Before switching-on of the actinic light and during the final 2 s of each step, a saturating light pulse of 6000 *μ*Em^−2^s^−1^ is applied during 0.6 s, followed by a dark period (actinic light off) of 1.4 s; measurements are recorded before and after the saturating pulses and after the dark periods, which correspond to *F*′, $F_{m}^{\prime }$ and $F_{0}^{\prime }$ respectively (see section 4.1). The second experiment (Exp2) considers an actinic light profile at 2000 *μ*Em^−2^s^−1^ during 10 minutes followed by a dark period of 60 minutes. Saturating pulses are applied at regular intervals during this experiment in order to measure *F*′, $F_{m}^{\prime }$ and $F_{0}^{\prime }$. The third experiment (Exp3) is optimally designed in order to improve upon the parameter estimation as described later in Section 4.4.

Our previous work [15] assumed a constant 1% variance of the fluorescence flux measurements. Here, we assume a variance model whereby a constant term (equal to 0.0015) is added to the 1% of the fluorescence fluxes, accounting for the sensitivity of the photomultiplier measuring the fluorescence signal. The utilization of a more realistic variance model for the measuring device may cause further practical identifiability problems.

### 2.3 Photosynthesis Rate

Out of the possible ways of measuring the photosynthesis rate, we consider measurements of the maximal rate of photosynthetic oxygen evolution at a specific actinic light using a Clark electrode (Hansatech, UK). In order to use PI measurements for parameter estimation, knowledge of the exact experimental light protocol is paramount, as established in Bernardi et al. [22]. Here, we consider PI measurements obtained from three separate experiments. Each experiment, involves exposing a dark adapted sample to two different light intensities over variable time periods (the exact protocols are reported in Table 2). The photosynthesis rate is measured at the end of each time period, thus providing a total of six experimental PI points. Sample 1 is to be included in the calibration data set, whereas the other two experiments are used for model validation only.

**Table 2:** PI experimental protocol. Light irradiance and corresponding time duration for the PI measurements in all three experiments.

	Sample 1		Sample 2		Sample 3	
irradiance [μEm^−2^s^−1^]	400	1500	100	750	250	3600
stage duration [s]	130	150	230	200	150	130
photosynthesis rate [$g_{{\text{o}}_{2}}{\text{g}}_{\text{chl}}^{-1}{\text{h}}^{-1}$]	4.10	5.58	1.22	5.18	2.58	7.22

### 2.4 Antenna Size Measurements

Antenna size measurements are used to study the saturation dynamics of the PSII. A LED pump and probe JTS10 fluorometer in fluorescence mode is used for the measurement. Fluorescence inductions are measured in the infrared region of the spectrum upon excitation with blue light at 450 nm. 3-(3,4-dichlorophenyl)-1,1-dimethylurea (DCMU) is added at a concentration of 80 *μ*M to prevent oxidation of the primary quinone acceptor Q_A_ [25, 26]. In the presence of this inhibitor, the half-saturation time constant of the fluorescence rise response is known to be inversely proportional to the so called functional antenna size (ASII) [27].

**Fig 1:**
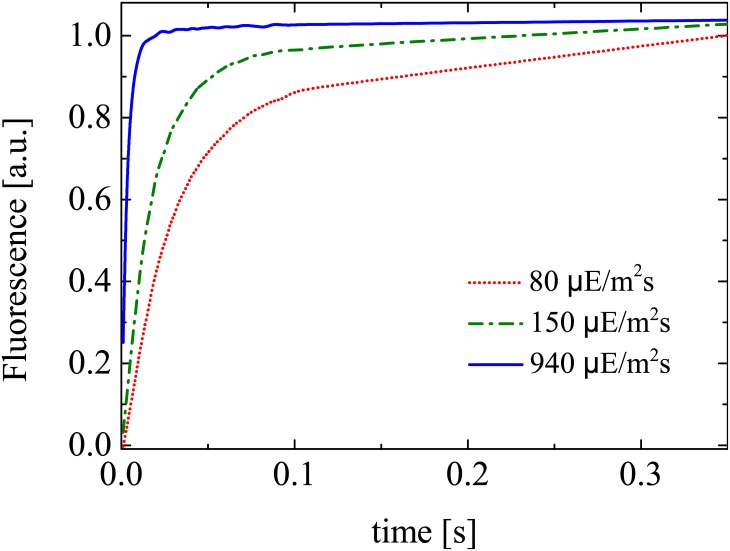
Antenna size experiment. Evolution of the fluorescence flux of PSII from dark-adapted acclimated cells treated with DCMU. The time required for reaching half of the maximum response is inversely proportional to ASII. The fluorescence flux is reported for three different actinic lights and normalised by the maximum flux value (a.u.: arbitrary units).

We consider antenna size experiments for five different actinic light intensities (45, 80, 150, 320, 940 and 2050 *μ*Em^−2^s^−1^) at 630 nm and four replicates are measured at each light intensity. Fig 1 shows a set of such experimental saturating curves. For each saturating curve the value of ASII is derived by fitting the data with a first order model, as further described in Section 3.2.

### 2.5 Numerical Aspects

Simulations of the fluorescence model are conducted in the modelling environment gPROMS (Process System Enterprise, gPROMS v 4.1, www.psenterprise.com/gproms, 1997–2016), together with the formulation and solution of parameter estimation and MBDoE problems. Parameter estimation is performed using maximum likelihood estimation and statistical confidence analysis [28], in order for the model predictions to match the measured fluorescence fluxes ($F_{m}^{\prime }$, $F_{0}^{\prime }$ and *F*′), and ASII and PI measurements. MBDoE optimises the actinic light profile and the measuring instants during a PAM experiment by minimizing the A-criterion [29], as other optimal criteria (namely D- and E-criteria) present numerical issues. The A-criterion aims to minimize the trace of the variance-covariance matrix of the parameter estimates to determine an information-rich experiment.

## 3 Model Development and Analysis

### 3.1 Modelling Chlorophyll Fluorescence

The model by Nikolaou et al. [15] predicts fluorescence fluxes by taking into account the state of the PSII and the activity of photoregulation. This model builds upon the well established state model by Han et al. [7] describing the state of the PSII in terms of open, closed and damaged RCII, and it introduces an activity equation describing the evolution of photoregulation in a semi-empirical manner (first-order process).

**Fig 2:**
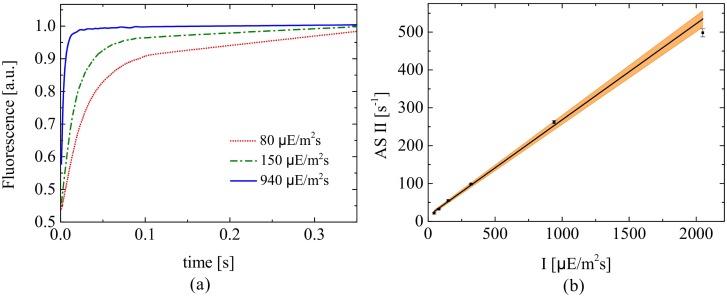
Constant actinic light PAM experiment. (a) Comparison between the predicted and measured fluorescence fluxes $F_{m}^{\prime }$ (triangles), $F_{0}^{\prime }$ (squares) and *F*′ (circles) in response to a constant light experiment. The grey-shaded area represents the light intensity. (b) Measured value of qNPQ, defined as $(F_{m}\;-\;F_{m}^{\prime })/F_{m}^{\prime }$, during the recovery phase of experiment Exp2 along with predicted values using different modelling assumptions. The dashed lines consider a first-order model to represent NPQ; the solid line considers the NPQ as the combined effect of two interdependent processes with different time scales.

This latter modelling assumption introduces an important limitation since, from a biological point of view, NPQ in algae is related to at least two distinct processes. The first one is a fast process involving the LHCSR protein, with a time constant of seconds for both its activation and relaxation [30]. The second one is related to the xanthophyll cycle and acts on a time scale of minutes. More specifically, zeaxanthin can have a complex effect on the NPQ activity as it both enhances the quenching effect of LHCSR and acts as an additional quencher [21]. In the model by Nikolaou et al. [15] the two interdependent NPQ processes are lumped into a single first order process. This approximation of the actual biological mechanism may lead to poor prediction of the fluorescence fluxes, for instance in experiments where the actinic light is kept constant for several minutes. This is illustrated in Fig 2(a), where the model predictions obtained with the parameter values established in [15], significantly deviate from the data generated by an experiment with a constant actinic light applied for 600 s, followed by a recovery period of 3600 s. Moreover, Fig 2(b) suggests that the recovery phase involves a two time-scale NPQ relaxation mechanism rather than a simple first-order process.

Given that a first-order process is not sufficient to accurately describe the actual photoregulation mechanisms, we propose the following, more complex dynamics:

$${\dot{\alpha }}_{F}=\xi_{F}\left(\alpha_{SS}-\alpha_{F}\right)$$ (1)

$${\dot{\alpha }}_{S}=\xi_{S}\left(\alpha_{SS}-\alpha_{S}\right)$$ (2)

$$\alpha_{SS}=\frac{I^{n}}{I_{\text{qE}}^{n}+I^{n}}$$ (3)

$$\eta_{qE}=\alpha_{F}\left({\bar{\eta }}_{qE}^{F}+\alpha {\bar{\eta }}_{qE}^{C}\right)+\alpha_{S}{\bar{\eta }}_{qE}^{S}.$$ (4)

The combined effect of LHCSR protein and zeaxanthin is apparent in Eq (4), where the parameters ${\bar{\eta }}_{\text{qE}}^{F}$ and ${\bar{\eta }}_{\text{qE}}^{S}$ represent the rates of the fast (LHCSR-related) and slow (zeaxanthin-related) NPQ processes, both relative to the rate of fluorescence. The parameter ${\bar{\eta }}_{\text{qE}}^{C}$ represents the enhancing effect of zeaxanthin in the quenching capability of the LHCSR protein. The activities of the fast and slow NPQ processes are described by the conceptual variables α*_F_* and α*_S_*, both modelled as first-order processes. Moreover, the reference α*_SS_* is the same in Eqs 1 and 2 since LHCSR- and zeaxanthin-related quenching are both triggered by low lumenal pH [21], yet with different time constant *ξ_F_* and *ξ_S_*. The reference α*_SS_* itself is modelled as a sigmoid (Hill) function, in agreement with experimental measurements of the NPQ index as function of *I* in [31], with *I*_qE_ and *n* representing the irradiance level at which half of the maximal NPQ activity is triggered (α*_SS_* = 0.5) and the sharpness of the switch-like transition, respectively. Overall, the NPQ dynamic is parametrized by ${\bar{\eta }}_{\text{qE}}^{F}$, ${\bar{\eta }}_{\text{qE}}^{S}$, ${\bar{\eta }}_{\text{qE}}^{C}$, *ξ_F_* and *ξ_S_*, instead of only two parameters in the original model [15]. The remaining model equations remain unchanged compared with [15], and are listed below for completeness:

$$F=S_{F}\sigma \Phi_{f}$$ (5)

$$\Phi_{f}=\frac{1}{\frac{A}{\Phi_{f}^{A}}+\frac{B}{\Phi_{f}^{B}}+\frac{C}{\Phi_{f}^{C}}}$$ (6)

$$\dot{A}=-I\sigma_{\text{PS}2}A+\frac{1}{\tau }B$$ (7)

$$\dot{B}=I\sigma_{\text{PS}2}A-\frac{1}{\tau }B+k_{r}C-k_{d}\sigma_{\text{PS}2}IB$$ (8)

1=A+B+C (9)

$$\sigma_{\text{PS}2}=\frac{\sigma }{N\nu }\Phi_{\text{P}}^{A}$$ (10)

$$\Phi_{P}^{A}=\eta_{P}\Phi_{f}^{A}$$ (11)

$$\Phi_{f}^{A}=\frac{1}{1+\eta_{P}+\eta_{D}+\eta_{qE}}$$ (12)

$$\Phi_{f}^{B}=\frac{1}{1+\eta_{D}+\eta_{qE}}$$ (13)

$$\Phi_{f}^{C}=\frac{1}{1+\eta_{I}+\eta_{D}+\eta_{qE}}.$$ (14)

Eq (5) defines the fluorescence flux, as a function of the total cross-section, $\sigma \;\;[{\text{m}}^{2}{\text{g}}_{\text{chl}}^{-1}]$; the fluorescence quantum yield, Φ*_f_* [−]; and a parameter depending on the characteristics of the PAM fluorometer and the chlorophyll content of the sample, *S_F_* [Vg_chl_m^−2^]. The fluorescence quantum yield, given in Eq (6), is the harmonic mean of the fluorescence quantum yields of open (*A*), closed (*B*) and damaged RCII (*C*). The dynamics of *A*, *B* and *C* are described according to the Han model [7] in Eqs (7)–(9), with the following parameters: the effective cross-section of the PSII, *σ*_PS2_ [m^2^ *μ*E^−1^]; the turnover rate, τ [s^−1^]; the damage rate constant, *k_d_*[−]; and the repair rate constant *k_r_*[s^−1^]. Eq (10) relates the effective cross-section, *σ*_PS2_ of the Han model with *σ*, effectively linking the state of the RCII with the fluorescence flux. It is important to note that while *σ*_PS2_ is a constant in the Han model, it becomes a function of the photoregulation activity (Eq 4), via the quantum yield of photosynthesis of an open RCII (Eqs 11 and 12). The parameter *N* represent the chlorophyll specific number of photosynthetic units $\left[\mu {\text{mol}}_{{\text{O}}_{2}}\;{\text{g}}_{\text{chl}}^{-1}\right]$, and *ν* is a stoichiometric factor reflecting the minimum theoretical value of 4 electrons for each dissociated water molecule per the water dissociation reaction (2H_2_O + 4e^−^ → O_2_ + 4H^+^). Eq (11) defines the quantum yield of photosynthesis of an open RCII. Finally, Eqs (12)–(14), define the quantum yield of fluorescence of the open, closed and inhibited PSUs, with the parameters *η_P_*, *η_D_* and *η_I_* representing, respectively, the rates of photoproduction, basal thermal decay in dark-adapted state and qI-quenching respectively. Overall, the maximum and minimum fluorescence fluxes $F_{m}^{\prime }$ and $F_{0}^{\prime }$ can be calculated from Eqs (5) and (6) by varying *A* and *B*. Specifically $F_{m}^{\prime }$ is obtained by imposing *A* = 0 and *B* = 1 − *C*, whereas $F_{0}^{\prime }$ by imposing *A* = 1 − *C* and *B* = 0. Moreover, the distinction between dark and light-adapted fluxes depends on the value of the variables *α_F_* and *α_S_*, with *α_F_* = *α_S_* = 0 for the dark-adapted state.

The improved representation of the NPQ mechanisms in Eqs (1)–(4) increases the complexity of the model in terms of the number of parameters from 13 to 16. The augmented parameter vector is as follows:

$$\theta =[\xi_{F},\xi_{S},n,I_{\text{qE}},k_{d},k_{r},N,\eta_{D},\eta_{P},{\bar{\eta }}_{\text{qE}}^{F},{\bar{\eta }}_{\text{qE}}^{S},{\bar{\eta }}_{\text{qE}}^{C},\eta_{I},\sigma ,S_{F},\tau ].$$

The parameter estimation problem is further complicated by the fact that the three recovery processes are now overlapping, while NPQ relaxation and damaged PSUs recovery were acting on distinct time scales in the original model [15].

### 3.2 Coupling Fluorescence with PI and ASII Measurements

The models (1)–(14) presents some identifiability issues [32] in the sense that not all of its parameters can be estimated uniquely when only fluorescence measurements are available. We note that, even in the original model [15], the parameters *η_D_*, *N*, *k_r_* and τ could not be uniquely estimated.

Based on the work by Falkowski and Raven [33], Bernardi et al. [22] derived an expression of the photosynthesis rate as:

$$P=\sigma \Phi_{P}I,$$ (15)

with Φ*_P_* the photosynthesis quantum yield [$\mu {\text{mol}}_{{\text{O}}_{2}}\;{\mu \text{E}}^{-1}$]. The units for p are [$\mu {\text{mol}}_{{\text{O}}_{2}}\;{{\text{g}}_{\text{chl}}}^{-1}\;{\text{s}}^{-1}$], which by definition is the chlorophyll-specific photosynthesis rate expressed in terms of O_2_ production. The fluorescence models (1)–(14), predicts the value of the realized quantum yield of photosynthesis, $\Phi_{\text{PS}2}=\frac{F_{m}^{\prime }-F^{\prime }}{F_{m}^{\prime }}$ in units of [$\mu {\text{mol}}_{{\text{e}}^{-}}\;{\mu \text{E}}^{-1}$], from which we then derive $\Phi_{P}=\frac{\Phi_{\text{PS}2}}{\nu }$ [34]. This way, the photosynthesis rate expression (15) does not introduce any additional parameters and conveniently couples PI measurements with fluorescence measurements in order to conduct parameter estimation. Although parameters *σ*, *S_F_* and *N* are structurally unidentifiable when using fluorescence measurements only, they become identifiable in adding PI measurements to the calibration set.

Note that fluorescence or PI measurements alone may only give information about the steady-state values of the fraction of open/closed RCII as a function of the light intensity, since the measurement frequency is not high enough to probe their dynamics. Based on such steady-state information it is only possible to estimate the ratio between *σ*_PS2_ and *τ*. In contrast, ASII measurements can provide information about this fast time scale, thereby providing a means of estimating the parameters *σ*_PS2_ and *τ* independently.

The addition of DCMU to a dark-adapted sample inhibits the transition *B* → *A*, by preventing oxidation of the primary quinone acceptor *Q*_*A*_. This way, the fluorescence induction curves shown in Fig 1 contain information about the dynamics *A* → *B* only. The time constant of this activation process can be obtained by fitting a first-order response model [6–8] for each light intensity.

**Fig 3:**
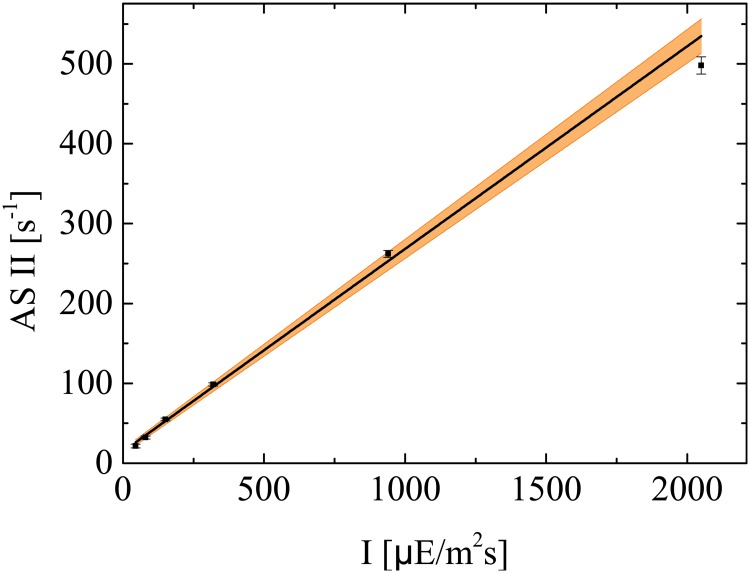
Antenna size experiment. Linear regression of ASII from the antenna size measurements at five different light intensities. The shaded area represents the confidence region of the linear regression.

The values of ASII for the different light intensities are reported in Fig 3 along with a regression fit. The standard deviations remain small with a maximum value of about 2.5% of the ASII value at 2050 *μ*Em^−2^s^−1^. The slope of the regression line is 0.25 ± 0.009[*μ*E^−1^m^2^] with an *R*^2^ value of 0.993.

Fig 3 validates the Han hypothesis of a linear correlation between ASII and the light intensity, and provides useful information to enhance the parameter estimation of the fluorescence model. Specifically, in our models (1)–(15) the antenna size corresponds to the product between the effective cross-section *σ*_PS2_ and the light intensity *I*, ASII = *σ*_PS2_ *I*. Therefore, the slope of the linear regression in Fig 3 represents the parameter of the Han model *σ*_PS2_ for a dark adapted sample (*α_F_* = *α_S_* = 0). Recall also that in the models (1)–(15), *σ*_PS2_ is related to the parameter *σ* via Eq (10) and is a function of the photoregulation activity. In particular, the time horizon of 0.35 seconds for the antenna size experiment is sufficiently short to prevent significant activation of the photoregulation mechanisms. One way to exploit the information obtained through the antenna size experiments consist of adding a virtual experiment to the calibration set, whereby the value of *σ*_PS2_ is a measured variable with a standard deviation of 0.009 μE^−1^ m^2^.

## 4 Model Validation Methodology

### 4.1 Step 1: Preliminary Identifiability Analysis

The model parameters are estimated using the two PAM experiments Exp1 and Exp2 described in Section 2.2, the PI measurements from Sample 1 described in Section 2.3 and the ASII measurements in Section 2.4. It can be established, e.g. by using a differential algebra approach as available through the software package DAISY [35], that all the parameters but *η_D_* are structurally identifiable with such a calibration set. In principle, one could estimate the parameter *η_D_*, representing the rate of basal thermal decay relative to the rate of fluorescence, based on the probability of thermal dissipation and the probability of fluorescence for a photon absorbed by a dark adapted RCII. Because this kind of measurements was not available to us, we set this parameter to *η_D_* = 5 subsequently, which is the mean of those *η_D_* values for which the computed fluorescence quantum yields are consistent with the data from Huot and Babin [36].

The results reported in Table 3 show that part of the estimated parameter values are not statistically meaningful. In particular:

- the parameters *k_r_* and *k_d_* may not be confidently estimated due to their large correlation with *η_I_*;
- the parameter ${\bar{\eta }}_{\text{qE}}^{F}$ has a very low sensitivity caused by the fact that the dynamics of LHCSR protein activation are much faster than the resolution of the PAM fluorometer.

**Table 3:** Preliminary model calibration. Parameter estimates along with their 95% confidence interval and t-values. The reference t-value is 1.65. The calibration set is comprised of Exp1, Exp2, ASII measurement and Sample 1 of PI measurements.

Parameter	Estimated value	95% conf. int.	t-value 95%	Units
*ξ_F_*	1.83 × 10^−1^	1.91 × 10^1^	**0.0096** *	s^−1^
*ξ_S_*	9.68 × 10^−4^	6.57 × 10^−5^	14.74	s^−1^
*I*_qE_	5.96 × 10^2^	4.18 × 10^1^	14.26	μEm^−2^s^−1^
*k_d_*	2.04 × 10^−6^	1.32 × 10^−6^	**1.55** *	−
*k_r_*	2.78 × 10^−5^	5.98 × 10^−5^	**0.46** *	s^−1^
*N*	5.31 × 10^−1^	8.61 × 10^−2^	6.16	$\mu {\text{mol}}_{{\text{O}}_{2}}\;{\text{g}}_{\text{chl}}^{-1}$
*n*	2.18 × 10^0^	1.74 × 10^−1^	12.54	−
*η_I_*	2.77 × 10^0^	1.45 × 10^0^	1.92	−
${\bar{\eta }}_{\text{qE}}^{F}$	8.17 × 10^0^	9.18 × 10^−1^	8.90	−
${\bar{\eta }}_{\text{qE}}^{S}$	1.92 × 10^1^	1.20 × 10^0^	16.02	−
${\bar{\eta }}_{\text{qE}}^{C}$	2.44 × 10^1^	3.32 × 10^0^	7.35	−
*η_P_*	1.14 × 10^1^	3.07 × 10^−1^	36.97	−
*σ*	7.79 × 10^−1^	1.19 × 10^−1^	6.55	$m^{2}{\text{g}}_{\text{chl}}^{-1}$
*τ*	8.45 × 10^−3^	1.07 × 10^−3^	7.87	s
*S*_*F*1_ ^a^	1.77 × 10^0^	2.72 × 10^−1^	6.53	Vg_chl_m^−2^
*S*_*F*2_ ^a^	1.97 × 10^0^	3.04 × 10^−1^	6.47	Vg_chl_m^−2^

^a^ *S*_*F*1_ refers to Exp1, *S*_*F*2_ refers to Exp2 experiment. The different values are due to different cell concentrations in the respective samples.

* an individual 95% t-value smaller than the reference t-value indicates that the available data may not be sufficient to estimate the parameter precisely

It is well known that even when a model is structurally identifiable, can measurements noise and other sources of uncertainty still hinder its practical identifiability [19, 37]. Since the parameters *k_d_*, *k_r_*, and *η_I_* are highly correlated, it is unlikely that generic experiments will provide the level of information that is needed to achieve statistically significant estimates for these parameters.

### 4.2 Step 2: Sensitivity Analysis

The sensitivity of a parameter *θ* with respect to a measured variable *y* can be computed as (*y*′ − *y*)/*Δθ*, where *y* and *y*′ are the measured variables calculated with the nominal and perturbed value of the parameter, respectively, and Δ*θ* is the parameter perturbation. By calculating the sensitivity of each parameter one can derive useful information about a model’s practical identifiability.

**Fig 4:**
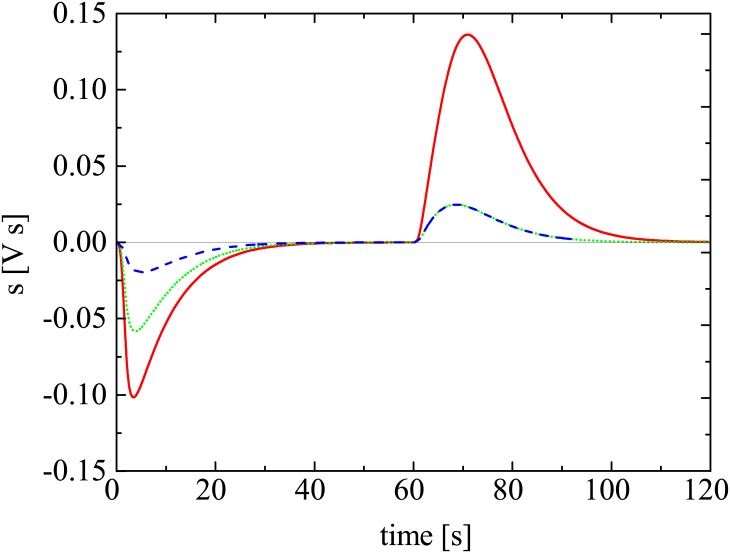
Sensitivity analysis. Sensitivity trajectories, *s*, of the fluxes $F_{m}^{\prime }$, $F_{0}^{\prime }$ and *F*′ with respect to the parameter *ξ_F_*. The red continuous line represents the sensitivity of $F_{m}^{\prime }$, the green dotted line represents the sensitivity of *F*′, and the blue dashed line represents the sensitivity of $F_{0}^{\prime }$. The light protocol used to obtain these curves is 60 seconds of actinic light at 2000 μEm^−2^s^−1^ followed by 60 seconds of dark. The nominal value of *ξ_F_* is 0.18 s^−1^.

We illustrate this technique by analysing the sensitivity of the parameter *ξ_F_* with respect to the measurement times of the fluorescence fluxes, considering a 5% increase in the value of *ξ_F_*. We simulate an experiment in which the actinic light is switched on for 60 s at 2000 *μ*Em^−2^s^−1^ and then switched off for another 60 s. The time-varying sensitivity profiles of $F_{m}^{\prime }$, $F_{0}^{\prime }$ and *F*′ with respect to the parameter *ξ_F_* are plotted in Fig 4. The sensitivities of the three fluxes pass by a maximum within a few seconds after the light switch occurs and then decrease exponentially to zero, confirming that *ξ_F_* may only be estimated if a measurement is taken shortly after the light has been switched on or off.

In a standard PAM experiment, like the two experiments used for calibration in the previous section, measurements are usually taken within 40 to 60 s after a variation in actinic light. It is therefore clear that the sensitivity of the measurements will usually be very low with respect to the parameter *ξ_F_* and the resulting estimate may not be confident. Instead, an informative (designed) experiment should ensure that some of the measurements are sampled when sensitivities of the measured variables are indeed maximal.

### 4.3 Step 3: Model-Based Design of Experiments

MBDoE is an effective methodology for designing experiments that contain maximal information based on a mathematical model, whose parameters are to be estimated. The experiment design is formulated as an optimisation problem wherein the decision variables correspond to time-invariant or time-varying inputs and the measurement times for the variables [18].

In practice, experimental designs often fail to take into account the relationship between the measured variables and the model parameters in order to effectively excite the system and to collect the measurements where they are the most informative. In other words, experimental protocols for model identification are not always designed in order to guarantee that the vector *y*(*t*) of measured output variables is maximally sensitive to the model parameters, as could be observed in the previous section for the parameter *ξ_F_*.

Here, the measured outputs are the three fluorescence fluxes $F_{m}^{\prime }$, $F_{0}^{\prime }$ and *F*′ that can be optimally sampled at 57 points during the experiment, with two consecutive measurements being separated by at least 40 s, a constraint posed by biological considerations. The experimental design variables consist of the corresponding measurement times, along with the actinic light profile as discretized on 20 constant light stages and whose durations are also decision variables. Note that the PAM fluorometer used to conduct the experiments can only apply discrete actinic light intensities of 0, 6, 13, 22, 37, 53, 70, 95, 126, 166, 216, 273, 339, 430, 531, 660, 825, 1028, 1287, 1594 and 1952 *μ*Em^−2^s^−1^. The total experimental horizon has been set equal to 2400 s and at the end of the experiment a dark period of 3 hours is added to better probe the slow dynamics of damaged RCII recovery. To summarise, the design vector includes: the measuring instants of the three fluorescence fluxes, the durations of actinic light stages, and the level of actinic light during each stage.

**Fig 5:**
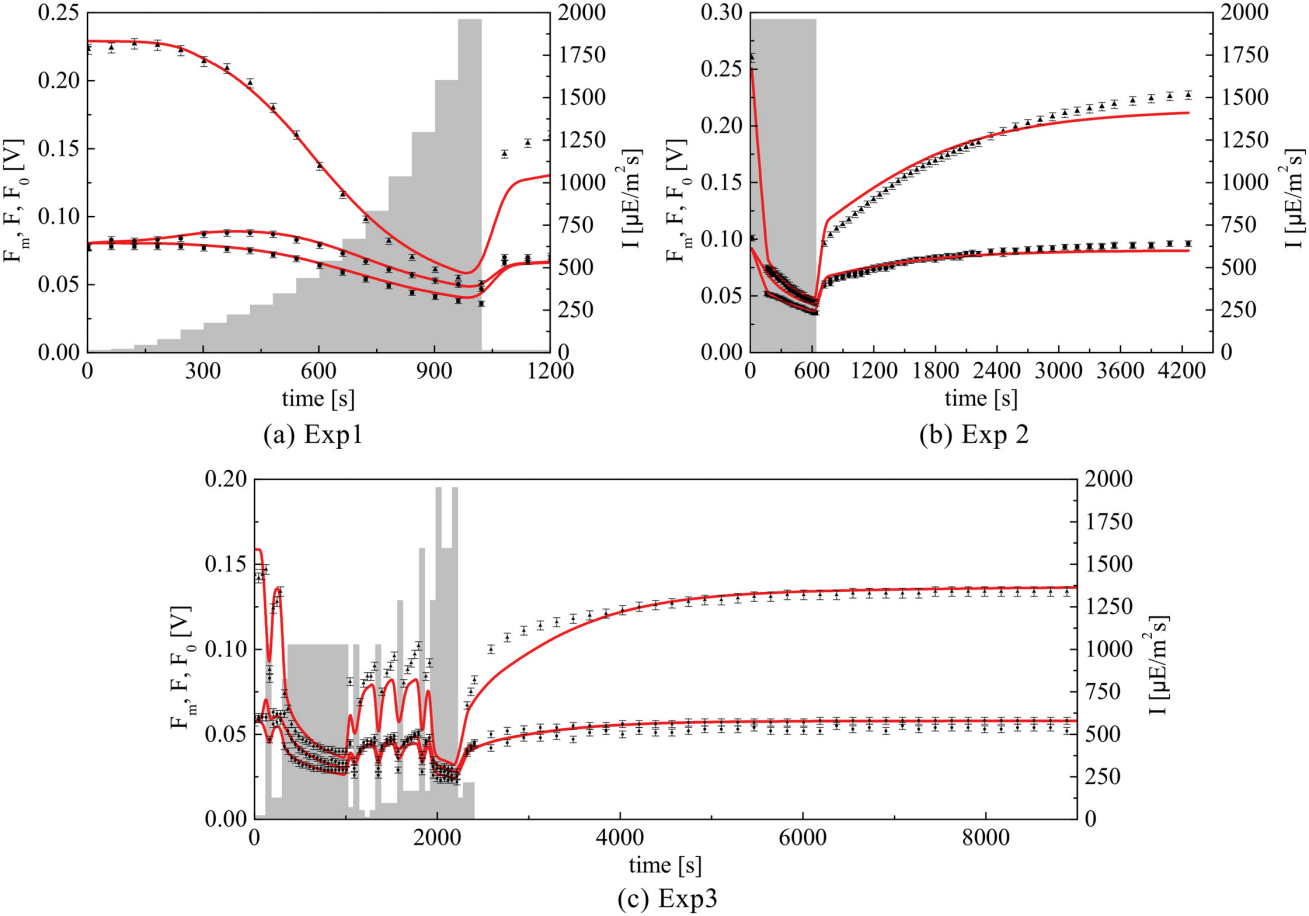
Final calibration results. PAM fluorescence profiles along with the model predictions corresponding to Exp1, Exp2 and Exp3 for the model parameters in Table 4.

The new fluorescence experiment designed with MBDoE, based on the preliminary parameters values obtained in Section 4.1 is shown in Fig 5(c), and referred to as Exp3 subsequently. Notice how MBDoE leads to a complex dynamic light protocol so as to excite the system in order to provide a highly informative input-output data set.

### 4.4 Step 4: Final Parameter Estimation

**Fig 6:**
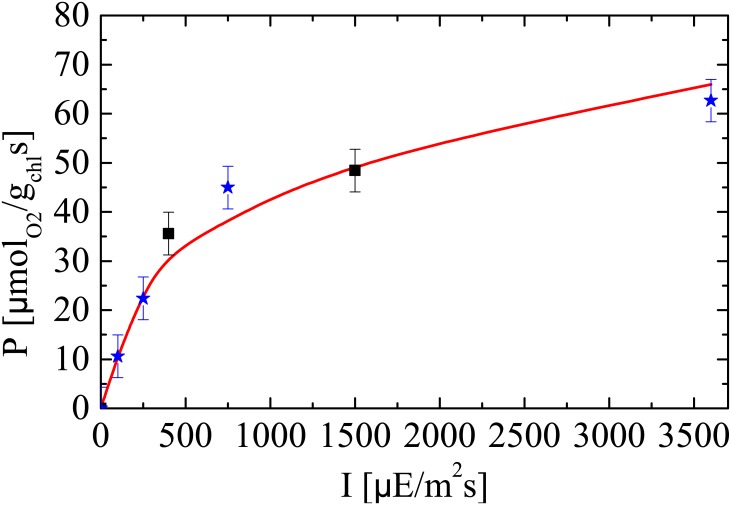
Experimental PI measurements along with model prediction. The solid line represents the model predicted PI curve; the black squares are the experimental data used for model calibration; and the blue stars represent the experimental data used for model validation.

The designed experiment (Exp3) is included along with Exp1 and Exp2 in the parameter estimation problem. The updated parameter estimates along with their 95% confidence intervals and t-values are listed in Table 4, and the fitting results of the model are presented in Figs 5 and 6.

**Table 4:** Final parameter estimation. Parameter estimates along with their 95% confidence interval and t-values. The reference t-value is 1.65. The calibration set is comprised of Exp1, Exp2, Exp3, Sample 1 of PI measurements and ASII measurements.

Parameter	Estimated value	95% conf. int.	t-value 95%	Units
*ξ_F_*	2.68 × 10^−1^	3.50 × 10^−2^	7.67	s^−1^
*ξ_S_*	1.32 × 10^−3^	6.97 × 10^−5^	18.88	s^−1^
*I*_qE_	5.95 × 10 ^2^	2.07×10^1^	28.76	μEm^−2^s^−1^
*k_d_*	9.95 × 10^−7^	2.67 × 10^−7^	3.73	−
*k_r_*	5.10 × 10^−5^	2.67 × 10^−5^	1.78	s^−1^
*N*	4.83 × 10^−1^	7.52 × 10^−2^	6.43	$\mu {\text{mol}}_{{\text{O}}_{2}}\;{\text{g}}_{\text{chl}}^{-1}$
*n*	2.40 × 10^0^	1.27 × 10^−1^	18.87	−
*η_I_*	1.41 × 10^1^	3.98 × 10^0^	3.54	−
${\bar{\eta }}_{\text{qE}}^{F}$	5.96 × 10^0^	4.98 × 10^−1^	11.95	−
${\bar{\eta }}_{\text{qE}}^{S}$	1.23 × 10^1^	5.75 × 10^−1^	21.35	−
${\bar{\eta }}_{\text{qE}}^{C}$	2.47 × 10^1^	1.69 × 10^0^	14.58	−
*η_P_*	1.04 × 10^1^	2.33 × 10^−1^	44.54	−
*σ*	7.33 × 10^−1^	7.50 × 10^−2^	6.84	$m^{2}{\text{g}}_{\text{chl}}^{-1}$
*τ*	6.95 × 10^−3^	7.50 × 10^−4^	9.26	s
*S*_*F*1_ ^a^	1.81 × 10^0^	3.01 × 10^−1^	6.82	Vg_chl_m^−2^
*S*_*F*2_ ^a^	2.06 × 10^0^	3.01 × 10^−1^	6.81	Vg_chl_m^−2^
*S*_*F*3_ ^a^	1.30 × 10^0^	1.90 × 10^−1^	6.82	Vg_chl_m^−2^

^a^ *S*_*F*1_ refers to Exp1; *S*_*F*2_ refers to Exp2; *S*_*F*3_ refers to Exp3 experiment. The different values are due to different cell concentrations in the respective samples.

The addition of experiment Exp3 now allows for the accurate estimation of both *ξ_F_* and *k_d_*, and besides, *k_r_* is also estimated in a statistically meaningful way. The rest of the parameters are also obtained with better confidence, thus demonstrating the effectiveness of MBDoE techniques. The estimated value of *η_I_* is significantly different from the one obtained in Table 3, which is not surprising considering its high correlation with the parameters *k_d_* and *k_r_*, and the fact that such parameters could not be properly estimated in the preliminary calibration in Section 4.1.

As shown in Fig 5, the model capability to represent the experimental data is generally very good, although some small mismatch can still be noticed. The first mismatch is related to the recovery phase of the three experiments, as the value of $F_{m}^{\prime }$ is underestimated by the model in Fig 5(a) and 5(c), whereas it is overestimated in Fig 5(b). Here, we have to consider that the three experiments were conducted using samples drawn from different cultures. Although the growth conditions were identical for all three cultures, the intrinsic biological variability of the system is likely to be responsible for the different responses in the experimental data.

The second mismatch is evident in Fig 5(c) between 1000 and 2000 s, where the predicted flux $F_{m}^{\prime }$ is underestimated during the low light periods. Although it is systematic this underestimation remains quite small. Future experiments will help understand whether this mismatch is related to structural inconsistency of the model, or if it can be attributed to biological variability or experimental issues.

The PI measurements simulated by the model along with the experimental values are reported in Fig 6. The black squares represent the data used for model calibration, whereas the blue stars are considered for model validation later in Section 4.5. The model shows a good agreement with the measured PI values used for model calibration (Sample 1). An interesting finding is that only two PI measurements are sufficient here to identify the parameter *N*, thus removing the need for time-demanding PI measurements.

Although some small discrepancies are observed, which may relate to the intrinsic variability of the microalgae sample, the model is able to reproduce experiments that are very different from each other, and statistically meaningful parameter estimates can be determined for all but one (*η_D_*) parameters.

### 4.5 Step 5: Model Validation

**Fig 7:**
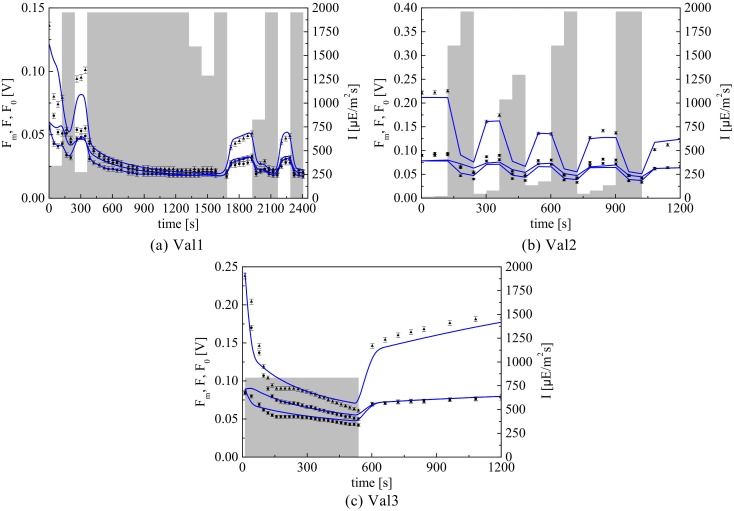
Model validation results. PAM fluorescence profiles along with the model predictions corresponding to Val1, Val2 and Val3, for the model parameters in Table 4.

Three additional PAM experiments are considered in order to validate the model. The validation set includes one constant light experiment (Val1) and two different variable light experiments (Val2 and Val3) where the sample is subject to complex actinic light profiles. The predicted fluorescence fluxes with the parameter estimates given in Table 4 along with the experimental data are shown in Fig 7. Moreover, two additional PI experiments are used for validation purposes, each consisting of two PI measurements, as shown in Fig 6.

The model predicts all three validation experiments in a very satisfactory way and is capable of capturing the variations in photosynthetic response triggered by different light dynamics. Moreover, the model gives accurate prediction of photosynthesis rate measurements. In particular, it is important to note that the model predictions remain accurate even for PAM protocols very different from the ones used in the calibration set, that is when the model is used for extrapolation. This confirms that the model provides an effective tool to predict PI curves without the use of classical measuring techniques that take time and resources to implement. The main discrepancy between the experiments and the model predictions can be observed in Fig 7(c) during the first 300 s of the experiment, which is similar in nature to the mismatch previously observed in Fig 5(c). However, the fits are generally good, thus confirming that the model is capable of accurate quantitative prediction for a wide range of PAM experiments. To our knowledge, no other mathematical model to date has been tested through such challenging experimental protocols or has demonstrated such a consistent prediction capability.

## 5 Conclusions

A mathematical model incorporating a detailed and biologically consistent representation of the NPQ mechanism has been proposed and validated using experimental data. The challenge of model identification has been tackled by introducing specific measurements, namely PI and antenna size measurements and by designing a tailored PAM protocol using MBDoE techniques. The results show that the model is able to predict chlorophyll fluorescence and photosynthesis rate with a good accuracy under a large variety of light conditions for N. Gaditana, thus paving the way towards a more reliable and realistic description of the effects of light dynamics on microalgae growth. Furthermore, the connection between PI curves and fluorescence has been verified practically, which could prove beneficial for reducing the experimental effort relative to PI curves experiments.

Future work will aim at moving up in the multiscale description of photosynthetic mechanisms and microalgae growth rate. This will involve incorporating a suitable description of photoacclimation phenomena into the model, following the work by Nikolaou et al. [10]. The focus will also be on combining the model with physics-based hydrodynamic and light attenuation models for full-scale photobioreactor simulation and optimisation [2].

## Supporting Information

S1 Dataset Experimental data. Excel file containing all the experimental data used in the paper.
(XLSX)
