# Correction: High-Fidelity Modelling Methodology of Light-Limited Photosynthetic Production in Microalgae

**DOI:** 10.1371/journal.pone.0156922

**Published:** 2016-06-03

**Authors:** Andrea Bernardi, Andreas Nikolaou, Andrea Meneghesso, Tomas Morosinotto, Benoît Chachuat, Fabrizio Bezzo

Fig 2 is incorrectly duplicated from Fig 1 and Fig 3. The authors have provided a corrected version here.

**Fig 2:**
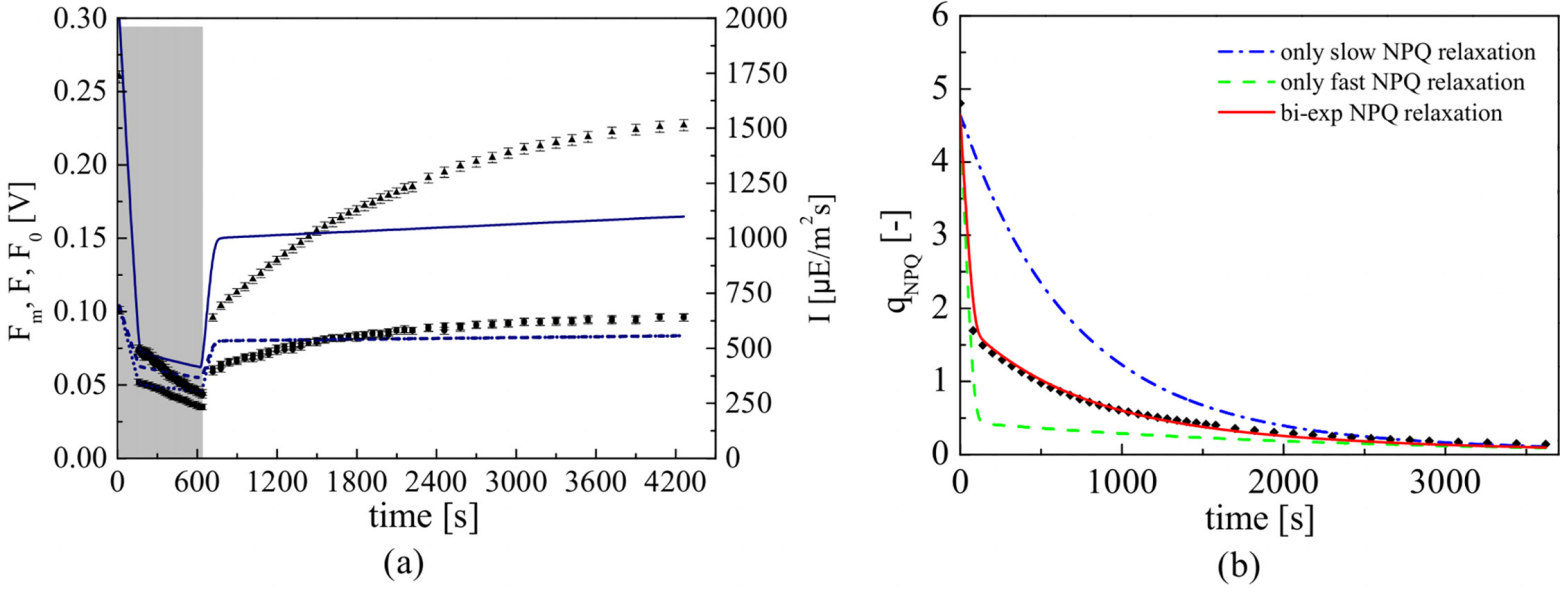
Constant actinic light PAM experiment. (a) Comparison between the predicted and measured fluorescence fluxes $F_{m}^{\prime }$ (triangles), $F_{0}^{\prime }$ (squares) and *F*′ (circles) in response to a constant light experiment. The grey-shaded area represents the light intensity. (b) Measured value of qNPQ, defined as $(F_{m}-F_{m}^{\prime })/F_{m}^{\prime }$, during the recovery phase of experiment Exp2 along with predicted values using different modelling assumptions. The dashed lines consider a first-order model to represent NPQ; the solid line considers the NPQ as the combined effect of two interdependent processes with different time scales.
